# ASGR1 Deficiency Promotes Liver Fibrosis by Enhancing Pro‐Inflammatory Arachidonic Acid Metabolism and ALP Secretion

**DOI:** 10.1002/iid3.70496

**Published:** 2026-08-20

**Authors:** Hui Zhu, Xin‐ping Huang, Kai You, Yan Chen, Peng‐hui Li, Jia‐wang Tao, Xiao‐rui Yu, Jie‐hui Xu, Guo‐sheng Xu, Yin‐xiong Li

**Affiliations:** ^1^ Clinical Biobank, National Infrastructures for Translational Medicine, Institute of Clinical Medicine Peking Union Medical College Hospital, Chinese Academy of Medical Sciences & Peking Union Medical College Beijing China; ^2^ Clinical Biobank Peking Union Medical College Hospital, Chinese Academy of Medical Science & Peking Union Medical College Beijing China; ^3^ State Key Laboratory of Complex, Severe, and Rare Diseases Chinese Academy of Medical Science & Peking Union Medical College Beijing China; ^4^ Institute of Development and Regeneration, China‐New Zealand Joint Laboratory on Biomedicine and Health Guangzhou Institutes of Biomedicine and Health, Chinese Academy of Sciences Guangzhou China; ^5^ University of Chinese Academy of Sciences Beijing China; ^6^ Institute of Biological and Medical Engineering, Guangdong Academy of Sciences Guangzhou China; ^7^ Division of Life Sciences and Medicine University of Science and Technology of China Hefei China; ^8^ Department of Blood Transfusion The Affiliated Guangdong Second Provincial General Hospital of Jinan University, Guangzhou Guangdong China

**Keywords:** alkaline phosphatase, ASGR1, biomarker, fibrosis, hepatic stellate cells, liver injury

## Abstract

**Background:**

Asialoglycoprotein receptor 1 (ASGR1), a hepatocyte‐specific receptor, represents a potential therapeutic target for hypercholesterolemia. However, liver safety risks of ASGR1‐targeted therapies remain poorly characterized. This study aims to investigate the impact of ASGR1 deficency on liver fibrosis and its underlying mechanisms.

**Methods:**

ASGR1 knockout (ASGR1^−/−^) mice and wild‐type controls were subjected to carbon tetrachloride (CCl_4_) administration or bile duct ligation (BDL) to induce liver fibrosis. Serum biomarkers, including alkaline phosphatase (ALP), alanine aminotransferase (ALT), and aspartate aminotransferase (AST), were measured. Transcriptomic analysis was performed to identify altered pathways. Primary mouse hepatocytes, HepG2 cells, and hepatic stellate cells (HSCs) were used for in vitro mechanistic validation.

**Results:**

ASGR1 deficiency significantly exacerbates liver fibrosis in both CCl_4_ ‐ and BDL ‐induced models and specifically induces an increase in serum alkaline phosphatase (ALP), which showed greater sensitivity than alanine aminotransferase (ALT) and aspartate aminotransferase (AST). Mechanistic studies showed that ASGR1 loss led to a significant increase in the transcription and secretion of hepatic ALP. Transcriptomic analysis revealed that ASGR1 knockout mice not only disrupted bile acid metabolism but also upregulated the synthesis of arachidonic acid metabolites (e.g., PTGES, a key enzyme involved in pro‐inflammatory eicosanoid synthesis) and the pro‐fibrotic factor TGFβ1, collectively accelerating fibrosis. Importantly, Supernatants from ASGR1^−/−^ primary mouse hepatocytes and HepG2 cells, as well as exogenous ALP, directly activated hepatic stellate cells (HSCs), promoting their activation and α‐SMA expression.

**Conclusions:**

ASGR1 deficiency aggravates liver fibrosis, which is likely mediated through the “ASGR1‐arachidonic acid‐ALP” axis that enhances pro‐inflammatory eicosanoid production and ALP secretion. These findings highlight the liver‐specific safety risk of ASGR1 as a therapeutic target for lipid disorders and provide mechanistic and translational references for clinical safety monitoring of ASGR1‐targeted drugs.

## Summary

- ASGR1 deficiency promotes liver injury by activating the arachidonic acid/TGFβ1 axis.
- Loss of ASGR1 in hepatocytes drives hepatic stellate cell activation through the specific secretion of ALP.
- Serum ALP is identified as a key biomarker for predicting fibrosis risk in ASGR1‐targeted therapies.

## Graphical abstract

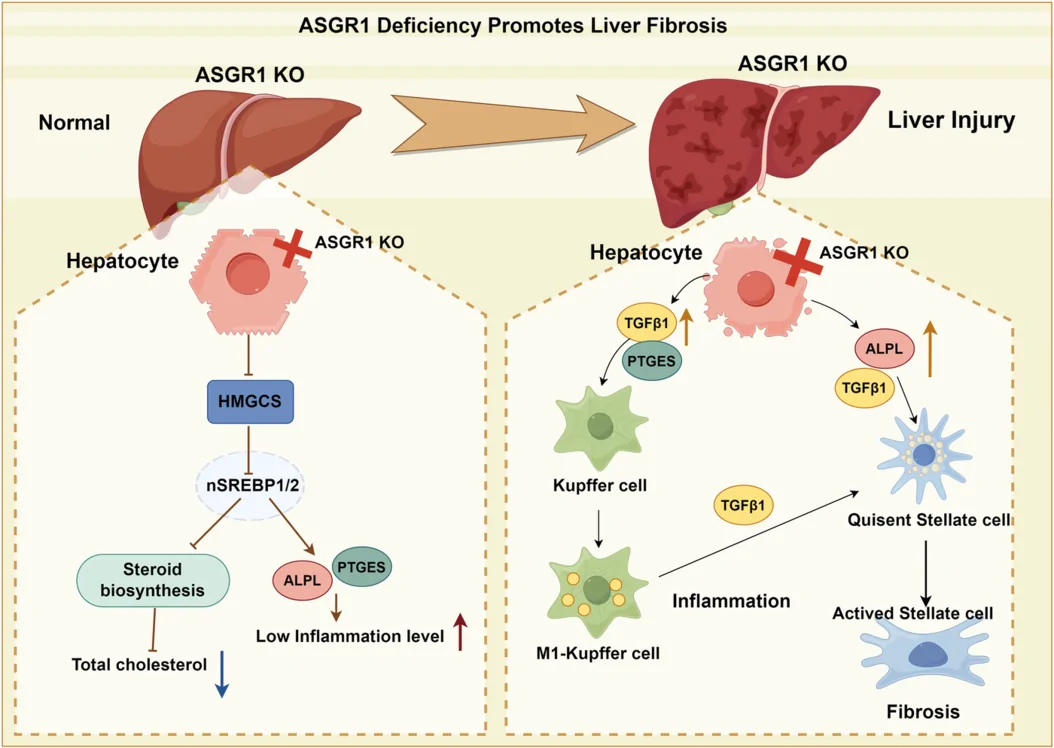

## 1 Introduction

The asialoglycoprotein receptor 1 (ASGR1), a receptor specifically expressed on the sinusoidal membrane of hepatocytes, is traditionally known for its role in glycoprotein homeostasis through the recognition and internalization of proteins bearing terminal galactose/N‐acetylgalactosamine (GalNAc) [1]. This function facilitates the clearance of senescent cells [2, 3] and platelets [4, 5, 6], and so forth. In recent years, ASGR1 has gained prominence as a compelling therapeutic target for hypercholesterolemia, following human genetic studies that linked its loss‐of‐function variants to favorable lipid profiles and reduced cardiovascular risk [7]. Subsequent work established that ASGR1 deficiency improves lipid homeostasis by modulating the INSIG1‐SREBP signaling pathway [8] and enhancing biliary cholesterol excretion [9], providing a solid rationale for the development of ASGR1‐targeting therapeutics.

However, the translational development of systemic ASGR1 inhibition is complicated by the protein's complex and context‐dependent roles in liver pathophysiology. Beyond its systemic benefits, ASGR1 appears integral to hepatic homeostasis, and its dysfunction may paradoxically promote liver injury. For instance, ASGR1 acts as a tumor suppressor in hepatocellular carcinoma, and its downregulation in human HCC is associated with poor prognosis [10, 11]. Conversely, ASGR1‐deficient porcine models exhibit unexplained liver damage [12]. Furthermore, in patients with liver fibrosis and corresponding mouse models, ASGR1 expression is downregulated, and its deficiency exacerbates endoplasmic reticulum stress and liver injury by upregulating GP73 [13]. These disparate findings underscore a critical safety consideration: the therapeutic inhibition of ASGR1 for cardiometabolic benefit may carry an inherent risk of provoking liver injury.

A major obstacle in evaluating and mitigating this risk is the pronounced knowledge gap regarding the specific effector molecules that mediate liver injury upon ASGR1 loss. The absence of a mechanism‐based, circulating biomarker to predict or monitor this adverse effect poses a significant challenge for the clinical translation of ASGR1‐targeted therapies. While prior research has elucidated cell‐autonomous responses in hepatocytes, intercellular communication from ASGR1‐deficient hepatocytes to pro‐fibrotic effector cells, particularly HSCs, remains largely unexplored. We hypothesized that a hepatocyte‐secreted factor links ASGR1 deficiency to HSCs activation and fibrogenesis. ALP, a classic clinical biomarker for cholestatic and biliary disease, emerged as a prime candidate.

Although ASGR1 has been implicated in the clearance of circulating ALP [14], its role in regulating the hepatic transcription and secretion of ALP—and, more importantly, ALP's potential function as an active profibrotic mediator in the context of ASGR1 dysfunction—has not been investigated. Thus, we hypothesized that ASGR1 deficiency drives liver fibrosis through the aberrant secretion of ALP, which in turn directly activates HSCs. This study aimed to investigate whether ALP serves not only as a biomarker but as a key mechanistic mediator in ASGR1 deficiency‐associated liver injury. Our findings seek to provide both fundamental insight into the pathological process and a practical tool for the safety monitoring of ASGR1‐targeted therapies.

## 2 Materials and Methods

### 2.1 Animal Experiments

All animal experiments were approved by the Experimental Animal Ethics Committee of the Guangzhou Institutes of Biomedicine and Health, Chinese Academy of Sciences, and conducted in accordance with the relevant ethical welfare guidelines and the core requirements of the ARRIVE Guidelines 2.0.

Male C57BL/6 mice (6‐8 weeks old) were used in this study. The mice were housed under specific pathogen‐free (SPF) conditions with a 12 h light/dark cycle, a controlled temperature of 18°C–22°C, and humidity between 40% and 60%. Standard chow and water were provided ad libitum. Asgr1‐knockout mice generated in Cyagen were provided by Xu Yingying [8]. All experimental and surgical operations complied with the animal ethical and welfare requirements.

#### 2.1.1 Sample Size, Randomization, and Blinding

Sample size was determined by a priori power analysis using G*Power3.1. Based on previous gene‐knockout studies reporting large effect sizes (Cohen's d = 1.2–1.8) [15, 16], a conservative effect size of d = 1.8 was adopted to ensure adequate statistical power. With α = 0.05 and power (1−β) = 0.8, the analysis indicated a minimum of 6 mice per group; 6 mice per group were used to account for potential attrition.

Mice were randomly assigned to WT, Asgr1^+/−^, and Asgr1^−/−^ groups using a random number table after 1 week of acclimatization.

All outcome assessments were performed in a single‐blind manner. Histopathological scoring, serum biochemistry, and Western blot quantification were conducted by independent investigators unaware of group allocation. Group codes were used during statistical analysis to avoid bias.

#### 2.1.2 Model Establishment

Subacute CCl_4_ injury: CCl_4_ (1 mL/kg, dissolved in mineral oil) was injected intraperitoneally twice weekly for 3 weeks. Mice were sacrificed 24 h after the last injection.

Chronic CCl_4_ injury: CCl_4_ was administered for 6 weeks; mice were sacrificed 72 h after the last injection.

BDL model: Mice were anesthetized, and the bile duct was double‐ligated for 21 days [17].

MASH model: The MASH mice were developed by Dr. Huang Xinping of our research group, who also provided the relevant samples. The method for establishing this model has been previously documented in the literature [18], Mice were fed a high‐fat, high‐cholesterol diet (TD.120528, Harlan Teklad) combined with weekly CCl_4_ (0.2 μL/g body weight, 20% CCl_4_ in olive oil) for 12 weeks. MASH‐related fibrotic lesions were observed after at least 12 weeks of feeding.

### 2.2 Cell Line and Cell Culture

The human liver hepatoma cell line HepG2 and the rat HSC line HSC‐T6 were maintained at 37°C in an atmosphere of 5% CO2. Both HepG2 and HSC‐T6 cells were cultured in Dulbecco's modified Eagle medium (DMEM) with high glucose content (Gibco), supplemented with 10% fetal bovine serum (FBS) (Gibco) and 1% nonessential amino acids (NEAA, Gibco).

Primary mouse hepatocytes and stellate cells were freshly isolated from 12‐week‐old male C57BL/6 J mice, with detailed culture conditions provided in the subsequent section on the isolation and culture of primary hepatocytes and non‐parenchymal liver cells.

### 2.3 Serum Biochemistry

Blood was obtained from the heart after overnight fasting, and plasma was further isolated via centrifugation at 3000 g for 15 min after the blood was left to stand for 2 h at room temperature. The serum levels of ALT, AST, ALP, TBA, T‐Bil, d‐Bil, and I‐Bil were measured using an automatic biochemical analyzer (7020; Hitachi) at Hunan Feng‐rui Biotechnology Co. (Hu Nan, China).

### 2.4 Quantitative Real‐Time RT‐PCR

A small amount of liver tissue was stored in liquid nitrogen and total RNA was extracted using TRIzol (Med Chem Express). Relative transcript levels were quantified by real‐time RT‐PCR using Bio‐Rad CFX96. Primers were designed using commercial Primer Express software (version 3.0; Perkin‐Elmer, Wellesley, USA). The housekeeping genes GAPDH and Tubulin were amplified in parallel for normalization. Primer sequences are summarized in Supporting Information Table S1.

### 2.5 Western Blotting

Cells or liver tissues were lysed with RIPA buffer (Beyotime) supplemented with a protease inhibitor cocktail, protease inhibitor PMSF, and phosphatase inhibitor. Centrifugation at 12,000 g for 15 min before ultrasound. Protein concentrations were determined using a BCA kit (Thermo Fisher Scientific, 23,225) according to the manufacturer's instructions. Samples were mixed with the 4X loading buffer (Gibco) and heated at 100°C for 10 min. The proteins were resolved by 10% SDS–PAGE in running buffer and electrophoretically transferred to PVDF membranes (Millipore) for 2 h. To block nonspecific binding with 5% skim milk powder, and the primary antibody was incubated at 4°C overnight. The next day, the membranes were washed with TBS for 5 min, three times, TBST (0.01% Tween) for 5 min. Then incubated with secondary antibody for 1 h at room temperature before washing with TBS for 5 min, three times, TBST (with 0.01% Tween) for 5 min. Finally, a chemiluminescence gel imaging system (Sage creation) and high‐sensitivity ECL Kit (Thermo Fisher Scientific) were used to detect the protein expression levels.

The following antibodies were used in the study: anti‐α‐SMA antibody (Abcam,1:1000); anti ‐GAPDH antibody (1:1000); anti‐ASGR1‐antibody (Proteintech 11739‐1‐AP,1:1000); anti‐BAX antibody (1:1000); anti‐COL1A1 antibody (Boster, catalog no. PA2140‐2, diluted 1:1000); anti‐ALPL antibody (ABConal,1:1000); anti‐TUBULIN antibody (Proteintech, catalog no.66031, diluted 1:1000). Goat anti‐rabbit IgG secondary antibody (Beyotime, catalog No. A0208, diluted 1:3000) was used. The antibodies used in this study are listed in Table 3.

### 2.6 Histological Examination

Liver samples were fixed overnight in 4% PFA and embedded in paraffin to prepare the paraffin sections of WT mice, Asgr1^+/−^and Asgr1^−/−^ mice. These sections were rewarmed at 65°C for 1 h, then washed with fresh paraxylene for 15 min, three times. The samples were washed twice with ethanol. The slides were gradient eluted with 95%, 80%, and 70% ethanol respectively before soaking in double‐distilled water. Next, peroxidase was inactivated with 3% H_2_O_2_ (diluted with methanol) for 30 min after washing twice with double‐distilled water. Hematoxylin–eosin and Sirius Red staining were performed using standardized clinical pathology protocols at the Histology Core.

For immunohistochemistry, the slides were placed in Tris‐EDTA buffer (pH = 9.0) and boiled with P100 for 5 min and P30 for 10 min. After natural cooling, the sections were washed thrice with TBS for 5 min. The samples were blocked with 10% goat serum (diluted in TBS) at room temperature for 1 h, then the samples were covered with the primary antibody at the indicated concentration overnight 4°C. The next day, the slides were washed with TBS three times for 5 min each time and incubated with the secondary antibody for 1 h at room temperature. A DAB Substrate Kit was used for staining. Safflower was used for nuclear staining for 5 min. Finally, the samples were rinsed with running water, placed in the 70%, 80%, 95%, and 100% ethanol for 5 min, sequentially washed with 100% ethanol and paraxylene for dehydration. After sealing, the slides were observed under a microscope. The primary antibodies used were as follows: anti‐α‐SMA antibody (Abcam,1:500); anti‐ALPL antibody (Abcam,1:200).

### 2.7 Immunofluorescence Staining

Cells were collected for 24 h and fixed in 4% PFA for 20 min. Then permeabilized by treatment with PBS containing 10% FBS and 0.02% Triton X‐100 for 15 min and washed by PBS for 5 min, three times. Thereafter, the cells were labeled with primary antibodies at 4°C overnight. The primary antibodies were detected using Alexa Fluor 488‐conjugated secondary antibodies (Life, USA). Nuclei were counterstained with DAPI (Sigma‐Aldrich, USA).

### 2.8 Isolation of Primary Hepatocyte and Non‐Parenchymal Liver Cell

Primary hepatocytes were isolated by two‐step collagenase perfusion, as described previously [19]. Briefly, primary hepatocytes were isolated from WT, Asgr1^+/−^, and Asgr1^−/−^ mice by 2‐step collagenase (Millipore Sigma) perfusion digestion and low‐speed centrifugation (50 g, 4°C), then viable hepatocytes were cultured in the collagen‐coated dishes in high DMEM containing 20% FBS and 2% penicillin and streptomycin (Life Technologies). The medium was changed to high DMEM supplemented with 10% FBS and 1% penicillin‐streptomycin after 4 h. Cell supernatants were collected for 24 h for subsequent cell culture experiments.

Primary non‐parenchymal liver cells were collected using a density gradient centrifugation system. Centrifugating at 700 g for 20 min at 4°C after collecting the supernatant from primary hepatocytes, the floating HSCs at the top of separation medium were collected and resuspended in DMEM supplemented with 15% FBS. Primary KCs were isolated in a manner similarly to HSCs, except for gradient centrifugation. HSCs isolated from the mice were not passaged and were cultured for 7–10 days after isolation.

### 2.9 ALP Activity Assay

Cells and supernatants were collected after 24 h, then cells were lysed with RIPA buffer before centrifugation at 500 g for 5 min at 4°C, and the supernatant was transferred to a new tube. The ALP activity of the medium supplemented with FBS and cell culture supernatant was determined using an Alkaline Phosphatase (AKP/ALP) Activity Assay Kit (Solarbio, BC2145), according to the manufacturer's instructions.

### 2.10 RNA‐Seq and Expression Analysis

Transcriptome sequencing was performed on liver tissues from CCI_4_‐induced acute liver injury and BDL‐induced liver injury mice as well as their respective controls. RNA‐seq relative data analysis of this subject was carried out by IGE Biotechnology Co. Ltd. (Guangzhou, China).

Furthermore, protein‐protein interactions pertinent to this study were identified using the STRING database (https://string‐db.org) for differential gene analysis, with visualizations enhanced using the Cytoscape software. Single‐cell gene expression databases for human liver injury models were obtained from the Gepliver (www.GepLiver.org).

### 2.11 Statistical Analyzes

Data are presented as the mean ± standard deviation (SD). Statistical analyzes were performed using GraphPad Prism, version 10 (GraphPad Software, San Diego, CA, USA). Normality was tested by Shapiro–Wilk test; variance homogeneity was tested by Levene test. Only after confirming that the data met the assumptions of parametric tests were subsequent statistical analyzes performed. One‐way analysis of variance (one‐way ANOVA) was used for comparisons among multiple groups. When there were significant differences between groups, Tukey's test was further used for post‐hoc multiple comparisons, and Bonferroni's method was used for multiple test correction to control the occurrence of false‐positive results. For non‐parametric data, Mann‐Whitney *U* test or Kruskal‐Wallis test with Dunn's post‐hoc test were applied. Statistical significance is indicated by *p ≤ 0.05, **p ≤ 0.01, and ***p ≤ 0.001.

## 3 Results

### 3.1 Expression Status of ASGR1 in Liver Tissues With Various Injuries or Diseases

**Figure 1:**
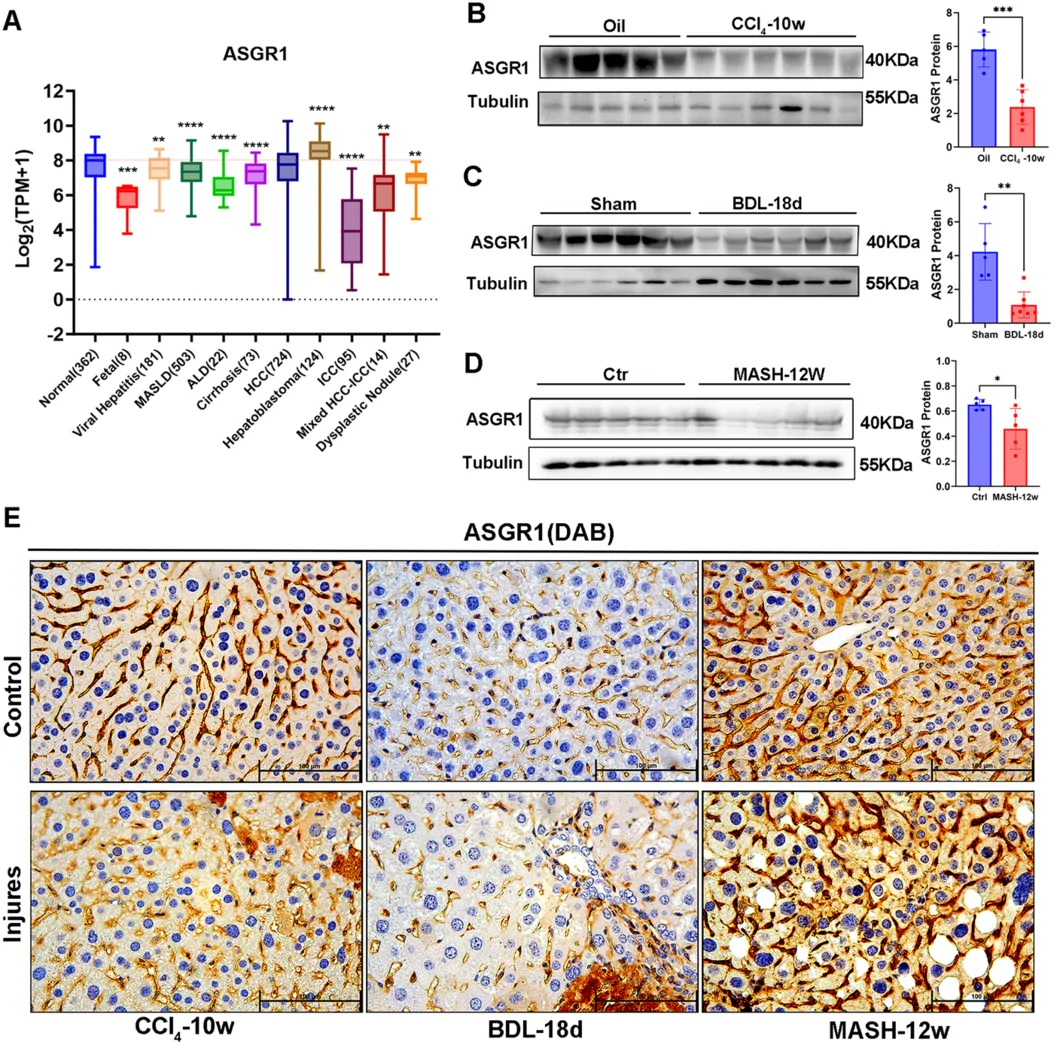
Expression status of ASGR1 in liver tissues with various injuries or diseases. (A) Gepliver database analysis shows the expression of ASGR1 in human liver damage tissues caused by different etiologies. (B–D) ASGR1 is down‐regulated in different mouse models of liver fibrosis. (E) Immunohistochemistry analysis of ASGR1 expression in CCI_4_, BDL and MASH model, which is mainly expressed on the sinusoidal membrane of hepatocytes. *p < 0.05, **p < 0.01, ***p < 0.001.

To investigate the expression of ASGR1 in different human liver injury tissues, we analyzed it using Gepliver database, a human liver single‐cell database [20]. The results showed that ASGR1 expression levels were lower than those in normal controls across various causes of liver injury, except for hepatoblastoma (Figure 1A).

To further explore the effects of different injury factors on ASGR1 expression in wild‐type mice, we established three liver injury models: chronic CCl_4_ administration for 10 weeks, BDL surgery performed 18 days post‐operation, and MASH induction over 12 weeks. Western blot analysis revealed that ASGR1 expression levels were significantly reduced in the livers of mice in all three liver injury models (BDL vs Sham, *p* = 0.0013; CCl_4_ vs Sham, *p* = 0.0004; MASH vs Ctr, *p* = 0.0327) (Figure 1B–D). Immunohistochemical results showed that ASGR1 was specifically localized on the side of the hepatocyte membrane facing the sinusoidal space. This distribution pattern was consistent with western blot results, particularly in the CCI_4_ injury model (Figure 1E).

### 3.2 Serum ALP Elevation Precedes Overt Liver Dysfunction in ASGR1‐Deficient Mice With Subacute Injury

**Figure 2:**
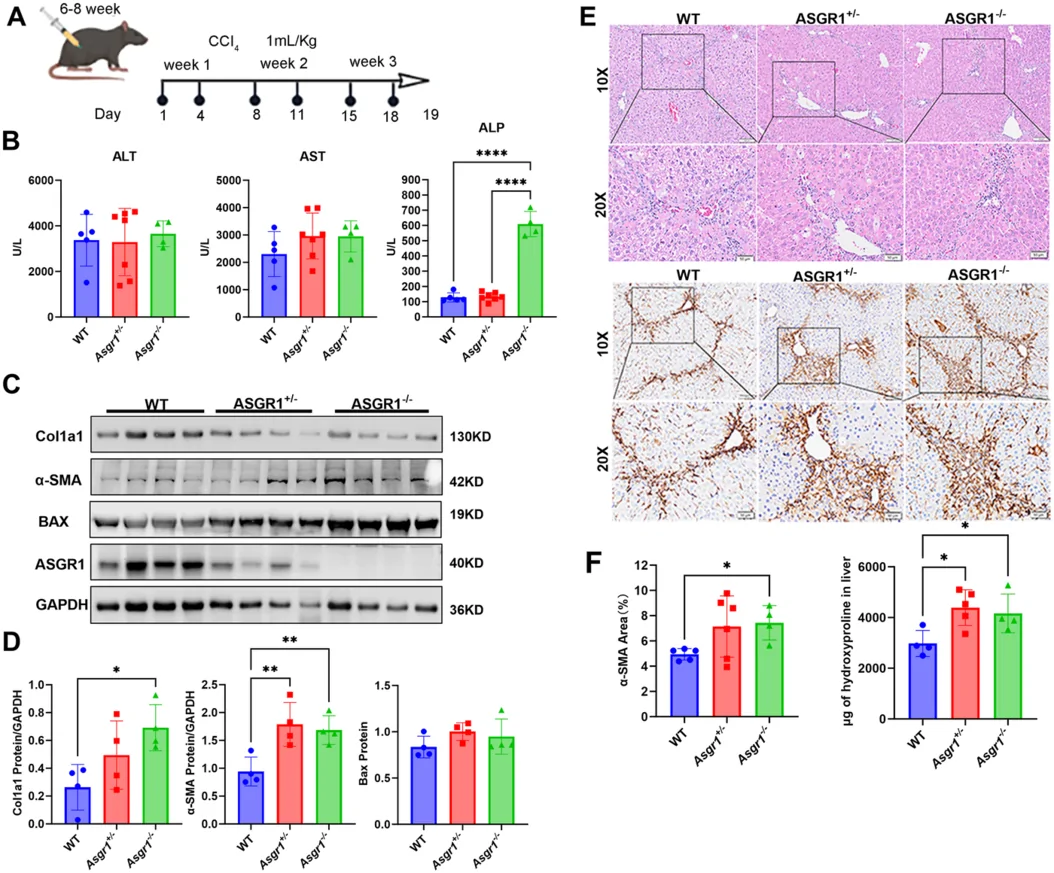
ASGR1^−/−^ mice exhibit worsened fibrosis in a model of subacute injury induced by CCl_4_. (A) Experimental design of chronic hepatotoxic CCl_4_‐induced in ASGR1‐deficient liver fibrosis model in 6–8 weeks male mice for 3 weeks (1 ml/kg per dose, two times a week, mice were sacrificed always 24 h after the last CCl_4_ administration). (B) Serum levels of liver injury indicators ALT, AST, and ALP. (C–D) Western blot analysis of α‐SMA, COL1A1 and Bax in the livers of mice, and the quantification of α‐SMA, COL1A1 and Bax in the livers of mice, GAPDH was used as internal reference for all proteins in the same sample (*n* = 4 per group). (E) Representative H&E and α‐SMA staining of liver sections from 8‐week‐old mice, scale bars: 50 μm, and 100 μm. (F) Statistical results of positive α‐SMA area and hydroxyproline assay for collagen (*n* ≥ 4 per group). All data are shown as mean ± SN (Means ± SN). *p < 0.05, **p < 0.01, ****p < 0.0001.

To assess the effects of ASGR1^−/−^ knockout mice on liver injury, we first established a CCl_4_‐induced subacute liver injury model (twice weekly for 3 weeks) (Figure 2A). The experimental results showed that serum ALT, AST, TBA, T‐Bil, d‐Bil, and I‐Bil were not significantly changed in the CCl_4_ subacute model (3 weeks), whereas ALP was strongly elevated in ASGR1^−^/^−^ mice (WT vs. Asgr1^−/−^, *p* < 0.0001; Asgr1^+/−^ vs. Asgr1^−/−^, *p* < 0.0001) (Figure 2B, Figure S1). α‐SMA and Col1a1 expression (α‐SMA: WT vs. Asgr1^+/−^, *p* = 0.0039; WT vs. Asgr1^−/−^, *p* = 0.008; Col1a1:WT vs. Asgr1^−/−^, *p* = 0.0128) α‐SMA–positive areas (WT vs Asgr1^−/−^, *p* = 0.0492) and hydroxyproline content were all increased (Hydroxyproline: WT vs. Asgr1^+/−^, *p* = 0.0039; WT vs. Asgr1^−/−^, *p* = 0.008) (Figure 2C–F). Thus, ASGR1 deficiency sensitizes mice to fibrosis with a preferential rise in serum ALP.

### 3.3 ASGR1 Deficiency Exacerbates Liver Injury and Fibrosis in Chronic Models

**Figure 3:**
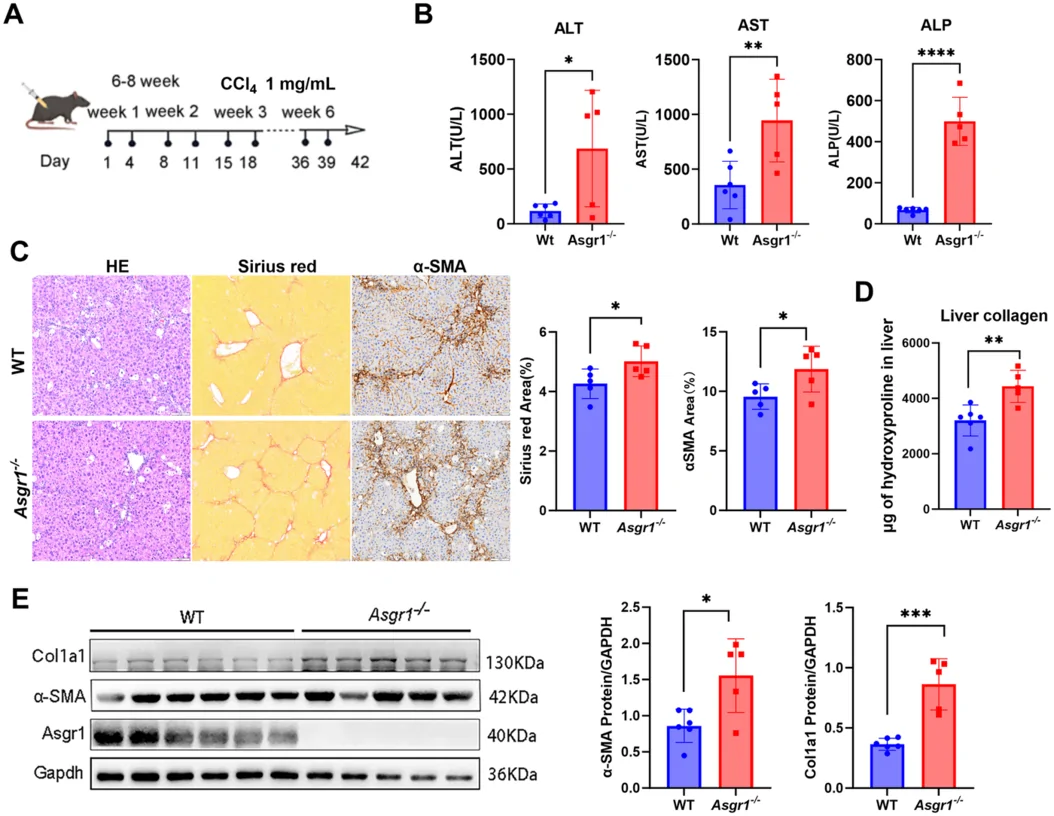
Liver injury and fibrosis increased in ASGR1 deficiency mice induced by CCl_4_ for 6 weeks. (A) Design of chronic liver injury model induced by CCl_4_ for 6 weeks (1 mL/kg per dose, two times a week, mice were sacrificed at 72 h after the last CCl_4_ administration). (B) Levels of ALT, AST, ALP in mouse serum of WT and Asgr1^−/−^ mice (WT, *n* = 6; Asgr1^+/−^, *n* = 5). (C) Representative H&E, immunohistochemistry, and Sirius red staining images, scale bar: 100 μm. Positive areas of α‐SMA and Sirius red were quantitatively analyzed (*n* ≥ 5 per group). (D) Statistical results of hydroxyproline assay for collagen (*n* = 5 per group). (E) αSMA and Col1a1 expressions in liver tissues were assessed by western blot, whereas GAPDH was used as an internal control. All data are presented as means ± SD, and *p < 0.05, **p < 0.01, ***p < 0.001.

To investigate the role of ASGR1 in chronic liver injury, the present study was performed to investigate the chronic liver injury modeling of CCl_4_‐induced and BDL‐induced chronic liver injury in ASGR1 knockout mice. As shown in Figure 3, during the CCl_4_‐induced chronic liver injury process, samples were collected 6 weeks after the onset of liver injury and 72 h after the final CCl_4_ injection (Figure 3A). The results showed that liver function indices, including ALT, AST, and ALP levels, were significantly elevated in ASGR1^‐/‐^ mice compared to WT mice (ALT, *p* = 0.0273; AST, *p* = 0.01; ALP, *p* < 0.0001) (Figure 3B). H&E and Sirius Red staining, α‐SMA immunohistochemical identification, and quantitative assessment of liver tissue hydroxyproline confirmed more severe morphological disruption of liver tissue, increasing inflammatory infiltration, enhanced α‐SMA expression, and increased collagen deposition in ASGR1^−/−^ mice compared to WT mice (Sirius Red, *p* = 0.045; α‐SMA area, *p* = 0.047; Liver hydroxyproline, *p* = 0.0058) (Figure 3C,D). In addition, liver tissue protein identification also confirmed that the expression of α‐SMA and col1a1 in the liver tissues of ASGR1 knockout mice was significantly higher than that in the WT model group (α‐SMA area, *p* = 0.0146; col1a1, *p* = 0.0003) (Figure 3E). These findings indicate that ASGR1^‐/‐^ mice have more severe liver injury following chronic liver injury.

**Figure 4:**
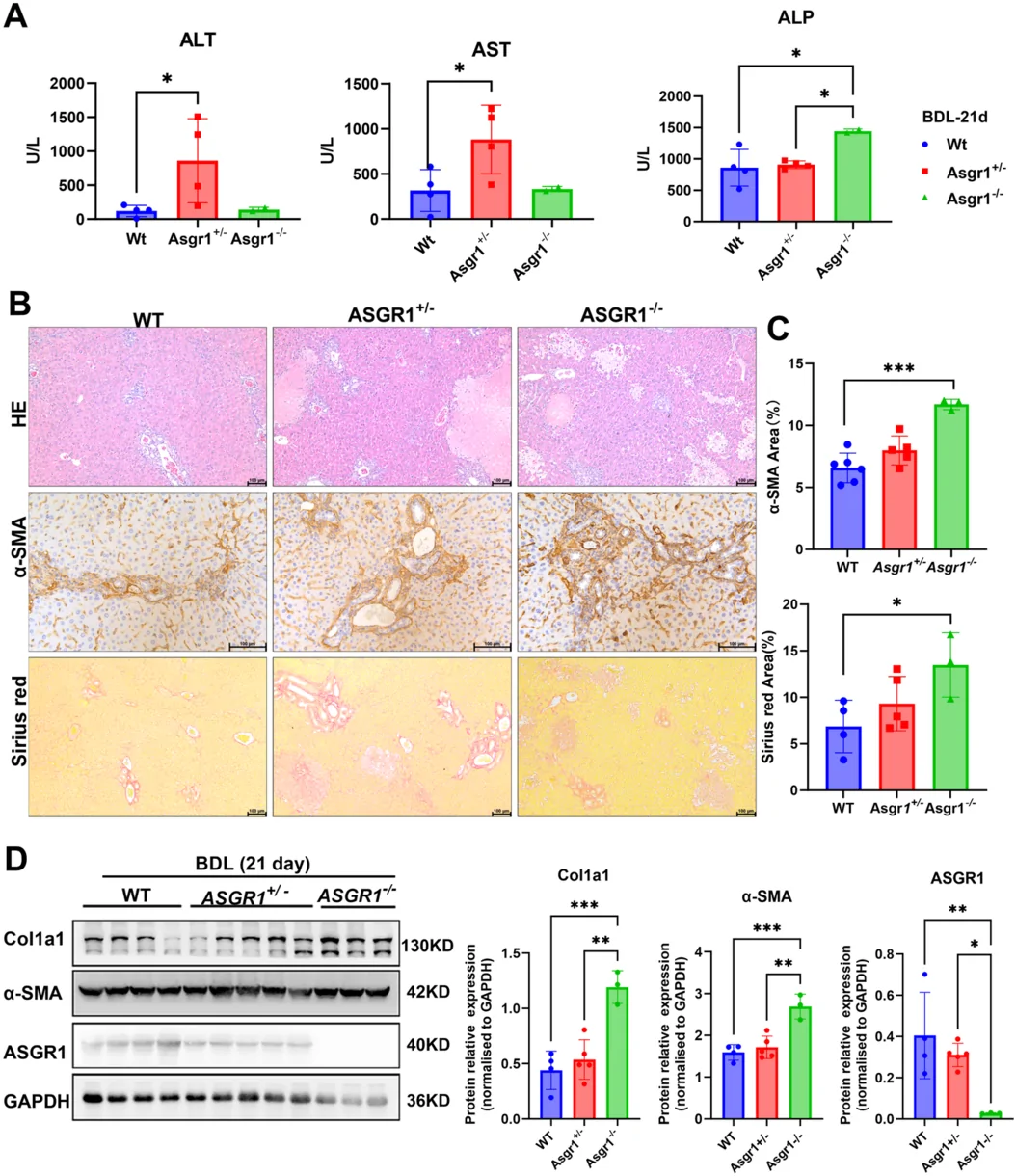
Asgr1^−/−^ mice exhibited worse liver injury and fibrosis in the BDL model as compared to WT. (A) The levels of serum ALT, AST and ALP in WT, Asgr1^+/−^, and Asgr1^−/−^ mice were measured after BDL for 3 weeks (WT, *n* = 4; Asgr1^+/−^, *n* ≥ 6; Asgr1^−/−^, *n* ≥ 3). (B,C) Representative H&E staining, α‐SMA immunohistochemistry staining and Sirius red staining (B), along with statistical results for positive area of α‐SMA and hydroxyproline levels (C) (WT, *n* = 6; Asgr1^+/−^, *n* = 5; Asgr1^−/−^, *n* = 3). (D) The expression of α‐SMA, Col1a1, and Asgr1 protein in liver tissues collected from WT, Asgr1^+/−^ and Asgr1^−/−^ mice after BDL for 3 weeks were analyzed by western blotting, while GAPDH was used as internal control. *p < 0.05, **p < 0.01, ***p < 0.001.

In addition, we also conducted research using a BDL model. Blood biochemical results showed that compared with WT mice, serum ALT and AST levels were significantly elevated in Asgr1^+/−^ mice (ALT: *p* = 0.0356; AST: *p* = 0.0437); however, serum ALP levels were specifically higher in Asgr1^−/−^ mice than in Asgr1^+/−^ mice and WT mice (ALP: WT vs. Asgr1^−/−^, *p* = 0.026; Asgr1^+/−^ vs Asgr1^−/−^, *p* = 0.038) (Figure 4A). Further IHC and WB analysis revealed that Asgr1^‐/‐^ mice exhibited the most significant increase in α‐SMA and Sirius red positive areas (WT vs Asgr1^−/−^: α‐SMA, *p* = 0.0002; Sirius red, *p* = 0.0443) (Figure 4B–D). These findings indicate that ASGR1 knockout exacerbates BDL‐induced liver fibrosis in mice, suggesting that ASGR1 plays a positive role in inhibiting fibrosis progression.

### 3.4 ASGR1 Knockout Activates Pro‐Inflammatory Arachidonic Acid Metabolic Pathway and Regulates Inflammatory Targets

**Figure 5:**
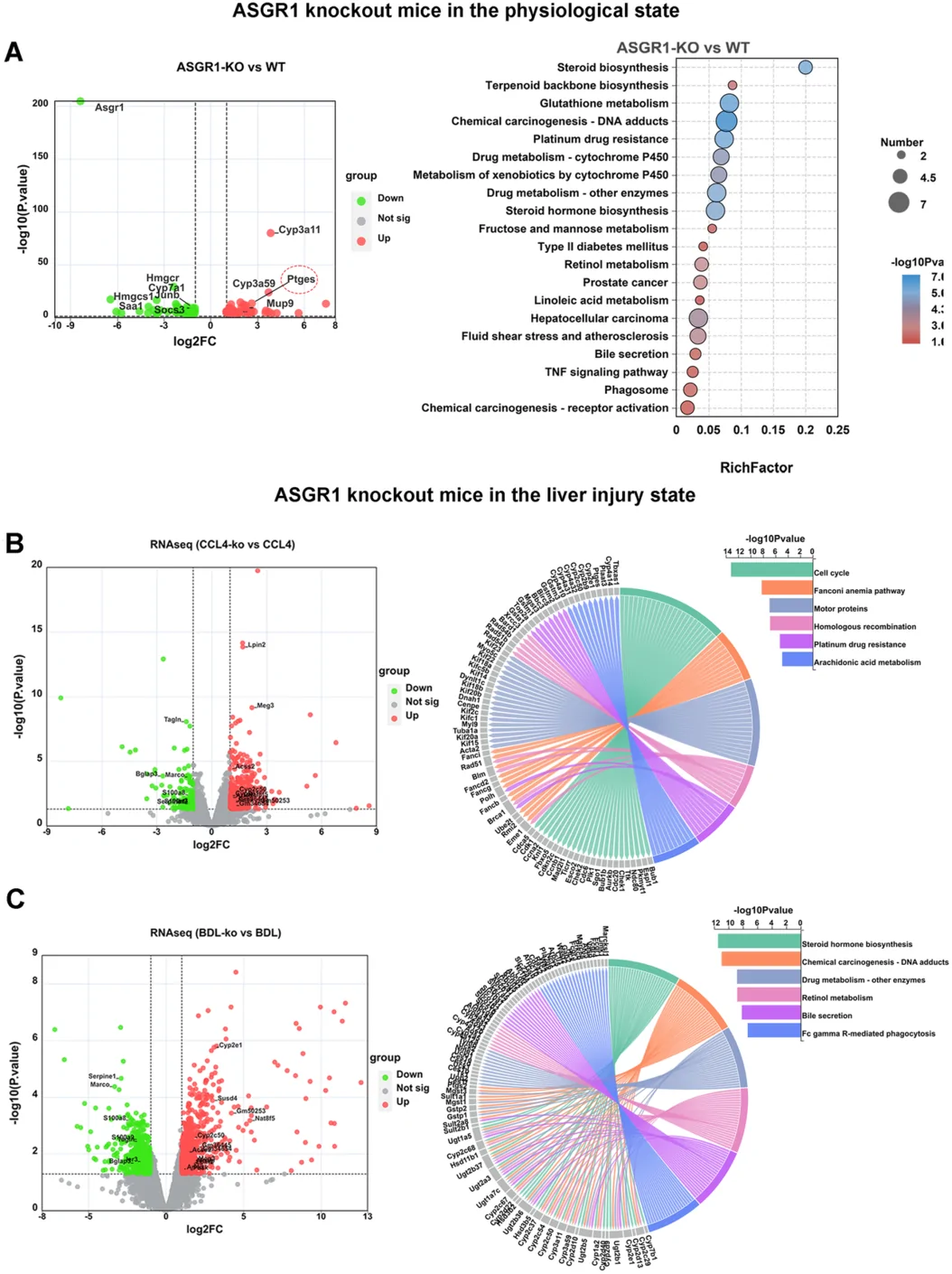
Transcriptomic profiling reveals the impact of ASGR1 knockout on hepatic gene expression under physiological and pathological conditions. (A–C) Transcriptomic profiling of the liver following ASGR1 knockout under (A) physiological conditions, (B) CCl_4_‐induced injury, and (C) BDL‐induced injury.

To elucidate the mechanisms underlying exacerbated liver injury in ASGR1 knockout mice, we first performed transcriptomic profiling (GSE178370) under physiological conditions [8]. The results showed that ASGR1 deficiency disrupted steroid synthesis and markedly upregulated PTGES, a key enzyme in pro‐inflammatory arachidonic acid metabolism, indicating a potential inflammatory pre‑activation state (Figure 5A).

**Figure 6:**
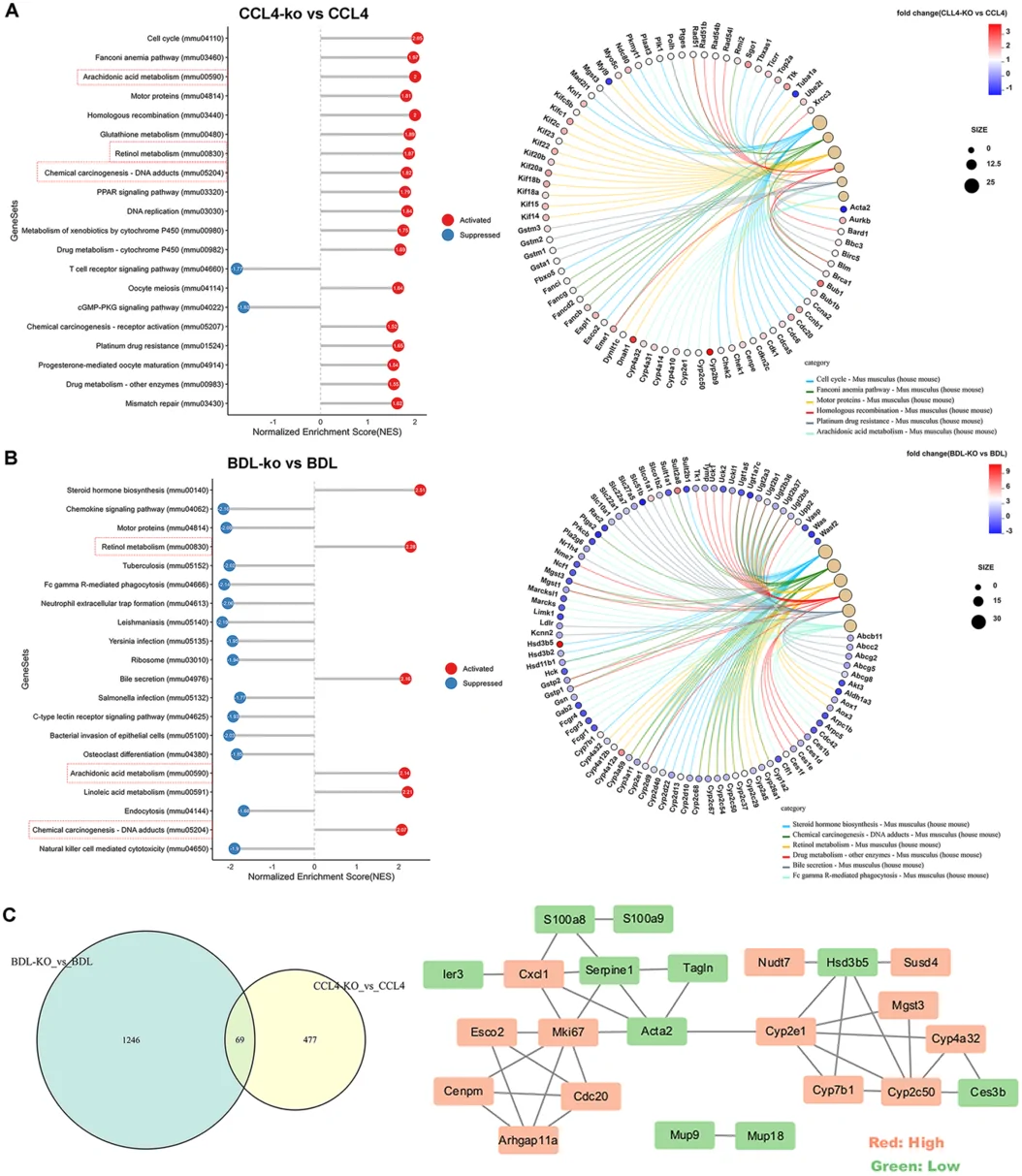
Transcriptomic analysis of ASGR1 gene knockout exacerbating liver damage. Liver transcriptomics analysis in ASGR1 knockout mice in CCl_4_‐induced liver injury (3 weeks) and bile duct ligation (BDL‐21day) models. (A,B) Show the results of gene set enrichment analysis (GSEA) comparing the enrichment levels of KEGG pathways in the two models. (C) Shows the transcriptomic sequencing data, including significantly differentially expressed genes (fold change > 2), the number of overlapping target genes, and their corresponding protein interaction networks. *p < 0.05, **p < 0.01, ****p < 0.0001.

RNA‑seq and gene set enrichment analysis (GSEA) in tissues from CCl_4_ ‑induced (3 weeks) subacute and BDL‑induced chronic liver injury models (Log_2_|FC | > 1, adj. *p* < 0.05) revealed that ASGR1 knockout significantly enriched arachidonic acid metabolism (e.g., PTGES, CYP2E1, CYP4A10), retinol metabolism, and chemical carcinogenesis‑related pathways in both models (Figure 5B,C, Figure 6A,B). Integrative analysis identified a perturbed steroid and oxidative metabolic network mediated by the CYP7B1–CYP2E1–CYP4A32 axis, consistent with elevated PTGES expression under physiological conditions (Figure 6C). Notably, ASGR1 knockout upregulated CYP2E1, a key cytochrome P450 enzyme involved in xenobiotic metabolism and oxidative stress, and enhanced the CYP7B1‑mediated bile acid bypass pathway [21, 22, 23, 24]. These changes collectively promote toxic bile acid accumulation, oxidative stress, and inflammatory activation, thereby accelerating liver injury.

We further systematically analyzed inflammation‑related targets in both injury models. A total of four common inflammatory targets were identified: Marco was significantly downregulated in both models (BDL: –3.32‑fold; CCl_4_: –1.19‑fold), which may regulate macrophage polarization toward an anti‑inflammatory M2 phenotype and contribute to hepatic immune modulation. Cxcl1, Lifr, and Nudt7 showed opposite regulatory patterns between models (e.g., Cxcl1 downregulated in BDL but upregulated in CCl_4_), suggesting injury‑specific inflammatory responses. In addition, 29 BDL‑specific inflammatory targets (including Ccl6, complement C6, Hmox1, Bcl6) were involved in complement activation, oxidative stress, and inflammatory cell recruitment, whereas 27 CCl_4_‑specific targets (including Socs2, Raet1d/e, Txnip) were associated with JAK–STAT signaling [25, 26], NK cell function, and NLRP3 inflammasome activation. Consistently, ASGR1 knockout significantly increased TGFβ1 levels in both subacute and chronic injury models (Supporting Information Figure S2), further promoting liver fibrosis by enhancing pro‑inflammatory factor secretion. Collectively, ASGR1 deficiency may promote liver fibrosis by regulating Marco‑mediated macrophage polarization, Cxcl1‑dependent neutrophil infiltration [27], and complement/cytokine signaling networks, which act synergistically with the ASGR1–arachidonic acid–ALP pathway.

### 3.5 ASGR1‐deficient Hepatocytes Secrete Elevated ALP That Directly Activates HSCs

**Figure 7:**
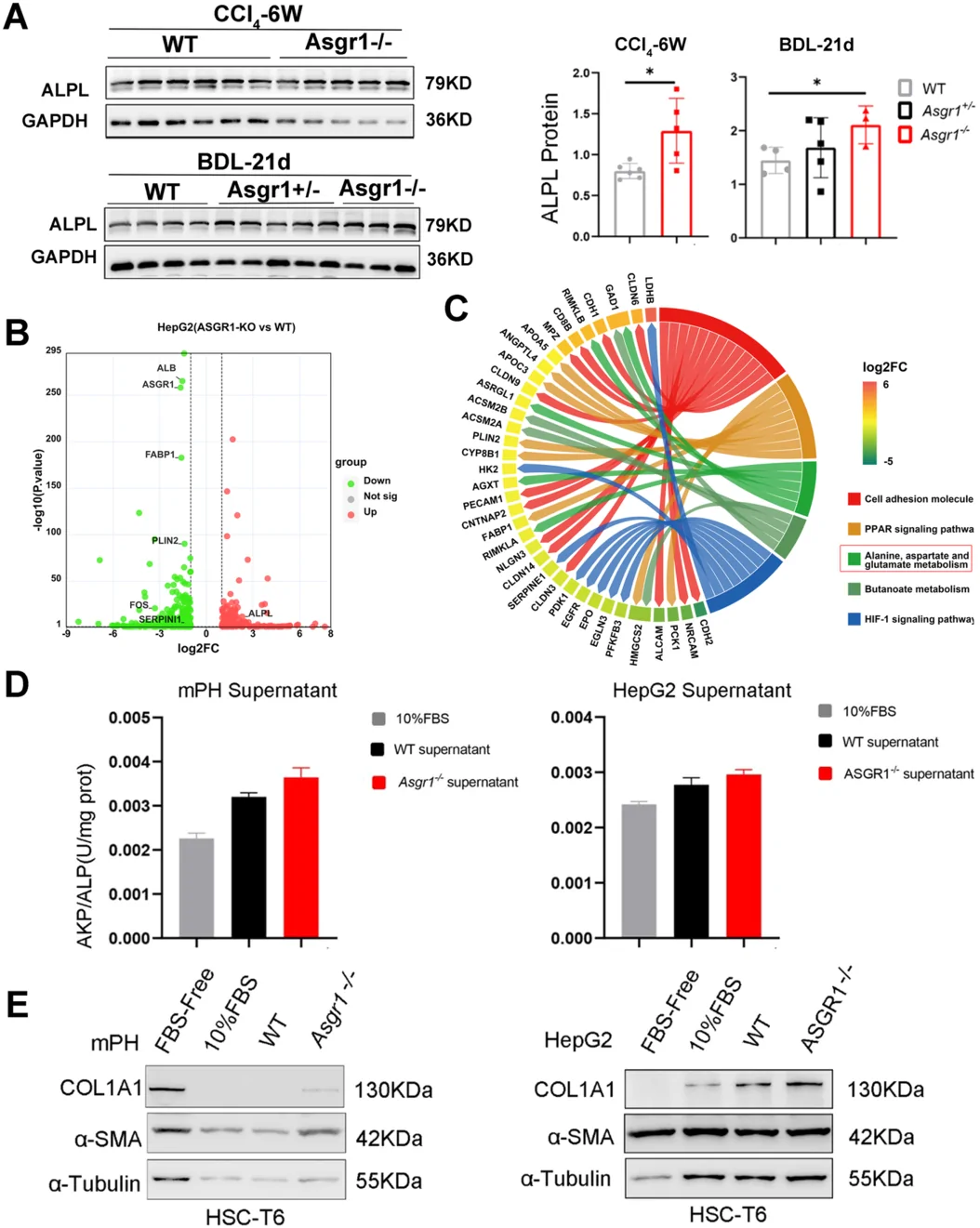
Hepatocytes with ASGR1 defects significantly overexpress ALP, and their culture supernatants promote the activation of hepatic stellate cells. (A) Protein expression of ALPL in liver tissue of ASGR1 knockout mice. (B) ALPL is significantly overexpressed in the transcriptome of HepG2 cells with ASGR1 knockout. (C) Effects of ASGR1 knockout on signaling pathways in HepG2 cells (ASGR1‐KO vs WT). (D) ELISA detection of ALP secretion in liver cells of ASGR1‐KO mice and HepG2 cells. (E) Effects of ASGR1‐KO primary liver cells and HepG2 cell culture supernatants on the expression of α‐SMA and COL1A1 in rat hepatic stellate cells (HSC‐T6). *p < 0.05, **p < 0.0001.

Previous studies have shown that ASGR1 negatively regulates ALP activity [14]. In this study, we found that serum ALP levels were significantly elevated in ASGR1 knockout mice in BDL, CCl_4_‐3ws, and CCl_4_‐6ws liver injury models. Although ALP is widely recognized as a marker of liver injury, its association with fibrosis remains unclear. In conclusion, we first examined ALPL expression in the liver tissues of mice with chronic liver injury, and the results confirmed that ASGR1 knockout indeed led to significantly high ALPL expression in the liver tissue (ALP: CCl_4_‐WT vs CCl_4_‐Asgr1^−/−^, *p* = 0.0156; BDL‐WT vs BDL‐Asgr1^−/−^, *p* = 0.0316) (Figure 7A).

To explore this mechanism in depth, we used CRISPR/Cas9 technology to knockout ASGR1 in HepG2 cells and performed transcriptomic sequencing analysis. As shown in Figure 7B‐C, the results showed that knockdown of ASGR1 significantly induced the high expression of ALPL and had a significant effect on the alkaline phosphatase signaling pathway.

Further functional experiments demonstrated that both primary mouse hepatocytes and HepG2 cells exhibited enhanced ALPL secretion after ASGR1 knockout (Figure 7D). By co‐culturing the supernatant from these cells with HSC‐T6 cells, we found that, compared to the normal control group, the supernatant from ASGR1‐knockout cells significantly promoted the expression levels of αSMA and Col1a1 in HSC‐T6 cells (Figure 7E).

This series of results support a functional link between hepatocyte ASGR1 loss, ALP secretion, and HSC activation.

## 4 Discussion

Our study identifies a novel mechanistic framework in which ASGR1 deficiency is strongly associated with accelerated liver fibrosis, likely mediated through aberrant hepatocyte‐derived ALP secretion and HSC activation. We provide comprehensive evidence supporting that ALP is not merely a bystander biomarker, but a functionally relevant mediator linking ASGR1 loss to HSC activation and fibrogenesis. This ASGR1–ALP–HSC regulatory axis provides a unified explanation for the liver injury phenotypes observed following ASGR1 inhibition and offers a clinically translatable safety monitoring strategy for ASGR1‐targeted therapies.

A major conceptual advance of this work is the redefinition of ALP from a passive indicator of cholestatic liver injury to an active pro‑fibrotic effector in the context of ASGR1 dysfunction. Although associations between ASGR1 inhibition and liver injury have been documented in previous studies [12, 13], the secretory factor that transmits pro‑fibrotic signals from ASGR1‑deficient hepatocytes to HSCs remained unknown. Our findings fill this critical gap. We demonstrate that ASGR1 deficiency transcriptionally upregulates ALPL expression and enhances ALP secretion in both mouse liver tissues and human HepG2 cells. Importantly, functional assays showed that conditioned medium from ASGR1‑knockout hepatocytes and exogenous ALP both directly promote HSC activation and α‑SMA expression. These observations support a mechanistic link rather than a definitive causal relationship (given the absence of in vivo rescue experiments), but strongly suggest that ALP acts as an important driver of fibrosis downstream of ASGR1 loss.

From an immunological perspective, transcriptomic‐based inflammatory target analysis further reveals that ASGR1 deficiency remodels the hepatic immune network, and this immunomodulatory effect synergizes with the pro‐fibrotic effect mediated by ALP to participate in the progression of liver fibrosis. Four common inflammation‐related targets were identified in both CCl_4_ and BDL models: Marco was consistently downregulated, which may regulate macrophage polarization toward the anti‐inflammatory M2 phenotype [28, 29]; Cxcl1, Lifr, and Nudt7 exhibited model‐specific opposite regulation, indicating injury‐dependent inflammatory responses. In addition, 29 BDL‐specific inflammatory genes (including Ccl6, complement C6, Hmox1, and Bcl6) and 27 CCl_4_‐specific genes (including Socs2, Raet1d/e, and Txnip) are involved in complement activation, inflammatory signaling, and NLRP3 inflammasome regulation [27]. These immunomodulatory effects synergize with the arachidonic acid–ALP pathway to jointly amplify the pro‐fibrotic microenvironment, further expanding the mechanistic understanding of liver fibrosis mediated by ASGR1 deficiency.

Our results are consistent with and extend recent findings by Zhang et al. (2024) [13], who reported that ASGR1 deficiency exacerbates acetaminophen‐induced acute and CCl_4_‐induced chronic liver injury in mice. Both studies confirm the hepatoprotective role of ASGR1, and the unique contribution of our study is the clear identification of ALP as a key functional intermediate linking ASGR1 loss, HSC activation, and fibrosis. This distinction is critical because it establishes the value of ALP as an applicable safety biomarker rather than a mere laboratory abnormality, providing a clear target for the safety monitoring of subsequent ASGR1‐targeted therapies.

To verify the liver specificity of elevated ALP in our models and rule out interference from ALP of other tissue origins, we performed multiple control analyzes. We confirmed that ASGR1 deficiency specifically upregulates hepatic ALPL expression without altering other ALP isoforms; the level of bone‐derived ALP (BALP) in ASGR1‐deficient mice did not change, further supporting the hepatocyte‐specific origin of ALP. Primary hepatocyte experiments further verified that ASGR1 deficiency can autonomously increase ALP secretion, reinforcing the hepatocellular origin of this phenotype and providing experimental evidence for ALP as a specific biomarker of liver injury.

Our findings carry profound translational implications for ASGR1‐targeted therapies. Although ASGR1 inhibition can effectively improve lipid profiles and reduce cardiovascular risk, its potential liver‐specific pro‐fibrotic risk cannot be ignored, presenting a mechanistic “double‐edged sword” effect. Notably, the early and significant elevation of serum ALP precedes the obvious changes in ALT and AST [30]. This feature supports its use as a sensitive pharmacodynamic safety marker for monitoring liver risk during the development of ASGR1‐targeted drugs, providing important reference for clinical medication safety [31, 32].

The pro‐fibrotic activity of ALP revealed in this study may not be limited to liver fibrosis, but extend to other fibrotic disorders. Previous studies have shown that tissue‐nonspecific ALP (TNAP) can promote cardiac fibrosis through the TGF‑β/Smad and ERK1/2 signaling pathways [33]; consistent with this, we observed increased TGFβ1 expression in ASGR1‐deficient fibrotic liver tissues, suggesting that ALP may widely participate in the fibrotic process of different tissues by activating conserved pro‐fibrotic signaling cascades. In addition, ALP can bind to collagen and regulate extracellular matrix remodeling [34], which provides another mechanism for the ASGR1–ALP axis to promote HSC activation and matrix deposition, further enriching the molecular pathway of ALP‐mediated fibrosis.

Several limitations of this study should be noted. First, this study used global ASGR1 knockout mice rather than hepatocyte‐specific knockout models, so the potential contribution of non‐parenchymal cells to liver fibrosis cannot be completely excluded. Second, human ASGR1 loss‐of‐function variants are predominantly heterozygous, and the clinical relevance of Asgr1 phenotypes needs further verification in long‐term studies and human cohorts to clarify their practical significance in clinical populations. Third, the HSC‑T6 cells used in this study are an immortalized rat cell line; verification in primary mouse or human HSCs will further strengthen our conclusions and improve the clinical applicability of the research results. Fourth, in vivo rescue experiments using ALP inhibitors or hepatocyte‐specific ALPL knockout are needed to formally establish the causal relationship of the ASGR1–ALP–HSC axis in liver fibrosis. Finally, the precise transcriptional cascade linking ASGR1 deficiency to ALPL upregulation (through arachidonic acid metabolism and PTGES) still requires further mechanistic dissection to improve the molecular details of the entire regulatory network.

In conclusion, this study demonstrates that ASGR1 deficiency is strongly associated with exacerbated liver fibrosis, likely mediated through increased hepatocyte ALP secretion, activation of arachidonic acid‐driven inflammatory pathways, and remodeling of the immune network. These findings highlight the key liver‐specific safety risks of ASGR1‐targeted therapies and support serum ALP as a sensitive and practical safety biomarker for clinical monitoring. Overall, this study deepens the mechanistic understanding of the role of ASGR1 in liver homeostasis, provides a reasonable theoretical framework for balancing the cardiovascular benefits and liver risks of ASGR1‐targeted therapies, and lays the foundation for the conduct of subsequent related studies.

## Author Contributions

**Hui Zhu:** writing – original draft, validation, project administration. **Xin‐ping Huang:** writing – review and editing, investigation, validation, data curation, formal analysis, project administration, software. **Kai You:** validation, conceptualization. **Yan Chen:** methodology, investigation. **Peng‐hui Li:** writing – review and editing, visualization, software. **Jia‐wang Tao:** validation, visualization. **Xiao‐rui Yu:** methodology, formal analysis. **Jie‐hui Xu:** validation, methodology. **Guo‐sheng Xu:** funding acquisition, writing – review and editing, resources. **Yin‐xiong Li:** writing – review and editing, resources, funding acquisition, conceptualization.

## Ethics Statement

All experiments in the present study involving animals were performed in accordance with the principles of the Declaration of Helsinki and were approved by the Ethical Review Committee of Animal Welfare (IACUC No. 2012036) of the Guangzhou Institute of Biomedicine, Chinese Academy of Sciences.

## Consent

All author(s) have approved the publication of this work.

## Conflicts of Interest

The authors declare no conflict of interest.

## Supporting information


Supporting File 1

Supporting File 2

## Data Availability

All raw data and code are available upon request.
