# Supplementary material for: ASGR1 Deficiency Promotes Liver Fibrosis by Enhancing Pro‐Inflammatory Arachidonic Acid Metabolism and ALP Secretion
Source: Immun Inflamm Dis. 2026 Aug 20;14(8):e70496. doi: 10.1002/iid3.70496 (PMC13491154; doi:10.1002/iid3.70496)
Supplement: Supplementary file 1 [file IID3-14-e70496-s002.pdf]

NOTE: Please save this file locally before filling in the table, DO NOT work on the file within your internet browser as changes will not be saved. Adobe Acrobat Reader (available free here) is recommended for completion.

The ARRIVE guidelines 2.0: author checklist

The ARRIVE Essential 10

These items are the basic minimum to include in a manuscript. Without this information, readers and reviewers cannot assess the reliability of the findings.

Item		Recommendation	Section/line number, or reason for not reporting
**Study design**	1	For each experiment, provide brief details of study design including:	
		a. The groups being compared, including control groups. If no control group has been used, the rationale should be stated. Three genotypes , including with wild-type (WT), Asgr1 heterozygous (Asgr1$^{+/-}$), and Asgr1 knockout (Asgr1$^{-/-}$) mice, were used for CCl_4 and BDL liver injury models. All analyses were compared with the WT group.	a. Methods 2.1.1
		b. The experimental unit (e.g. a single animal, litter, or cage of animals). Individual male C57BL/6 mouse.	b. Methos 2.1
**Sample size**	2	a. Specify the exact number of experimental units allocated to each group, and the total number in each experiment. Also indicate the total number of animals used. Per group: n = 6 (WT, Asgr1$^{+/-}$, Asgr1$^{-/-}$); total ≥ 54 mice.	a. Methods 2.1
		b. Explain how the sample size was decided. Provide details of any *a priori* sample size calculation, if done. A priori power analysis (G*Power 3.1); effect size d = 1.8, α = 0.05, power = 0.8.	b. Methods 2.1.1
**Inclusion and exclusion criteria**	3	a. Describe any criteria used for including and excluding animals (or experimental units) during the experiment, and data points during the analysis. Specify if these criteria were established *a priori*. If no criteria were set, state this explicitly. Inclusion: 6–8-week-old male SPF-grade C57BL/6 mice; Exclusion: unhealthy or deceased animals. Criteria predefined before experiments.	a. Methods 2.1
		b. For each experimental group, report any animals, experimental units or data points not included in the analysis and explain why. If there were no exclusions, state so. During experiments, all mice in the CCl_4-3w group completed modeling successfully; two Asgr1^{-}/$^{-}$mice died unexpectedly under anesthesia during blood collection, leaving n=4 for serum analysis. One mouse died during modeling in the CCl_4-6w group. Three Asgr1^{-}/$^{-}$ mice in the BDL group died from severe liver injury and cholestasis, leaving n=3.	b. Methods 2.1
		c. For each analysis, report the exact value of *n* in each experimental group. WT: n = 5–6; Asgr1$^{+/-}$: n = 5–6; Asgr1$^{-/-}$: n = 3–5.	c. Results 3.2-3.5
**Randomisation**	4	a. State whether randomisation was used to allocate experimental units to control and treatment groups. If done, provide the method used to generate the randomisation sequence. Mice were randomised using a random number table	a. Methods 2.1.1
		b. Describe the strategy used to minimise potential confounders such as the order of treatments and measurements, or animal/cage location. If confounders were not controlled, state this explicitly. Cage allocation and littermates were randomized.	b. Methods 2.1.1
**Blinding**	5	Describe who was aware of the group allocation at the different stages of the experiment (during the allocation, the conduct of the experiment, the outcome assessment, and the data analysis). Allocation: unblinded; Surgery: single-blind; Outcome assessment: double-blind; Data analysis: double-blind.	Methods 2.1.1

**Outcome measures**	6	a. Clearly define all outcome measures assessed (e.g. cell death, molecular markers, or behavioural changes).<br>Liver injury: assessed by serum biochemical markers including ALT, AST, ALP, TBA, and total/direct/indirect bilirubin.<br>Liver fibrosis: evaluated via Sirius Red staining and hepatic hydroxyproline content.<br>HSC activation: determined by the expression of α-SMA and col1a1.<br>b. For hypothesis-testing studies, specify the primary outcome measure, i.e. the outcome measure that was used to determine the sample size.<br>Primary outcome: 1) Liver fibrosis: sirius red staining and hepatic hydroxyproline content. 2) HSCs activation: α-SMA expression.	a. Results 3.1-3.5<br>b. Results 3.1-3.5
**Statistical methods**	7	a. Provide details of the statistical methods used for each analysis, including software used. One-way ANOVA + Tukey's post hoc test; non-parametric tests if required; GraphPad Prism 10.<br>b. Describe any methods used to assess whether the data met the assumptions of the statistical approach, and what was done if the assumptions were not met.<br>Shapiro–Wilk normality test, Levene's homogeneity test; non-parametric tests applied if violated.	Methods: 2.1.1 and 2.11
**Experimental animals**	8	a. Provide species-appropriate details of the animals used, including species, strain and substrain, sex, age or developmental stage, and, if relevant, weight.<br>Male C57BL/6 mice, 6–8 weeks old, body weight 24–28 g.<br>b. Provide further relevant information on the provenance of animals, health/immune status, genetic modification status, genotype, and any previous procedures.<br>All mice were maintained under SPF conditions on a C57BL/6 background, including wild-type (WT), Asgr1 heterozygous ($Asgr1^{+/-}$) and global Asgr1 knockout ($Asgr1^{-/-}$) mice, which were generated and provided by Cyagen Biosciences..	Methods 2.1
**Experimental procedures**	9	For each experimental group, including controls, describe the procedures in enough detail to allow others to replicate them, including:<br>a. What was done, how it was done and what was used.<br>CCl_4: 20% in mineral oil, 1 mL/kg, i.p., 2×/week;<br>BDL: double ligation of common bile duct; sham: laparotomy only.<br>b. When and how often. CCl_4: 3 weeks (subacute), 6 weeks (chronic); BDL: 21 days.<br>c. Where (including detail of any acclimatisation periods).<br>SPF animal facility; 1-week acclimatisation.<br>d. Why (provide rationale for procedures).<br>CCl_4 and BDL are well-established liver fibrosis models.	Method 2.1-2.1.2
**Results**	10	For each experiment conducted, including independent replications, report:<br>a. Summary/descriptive statistics for each experimental group, with a measure of variability where applicable (e.g. mean and SD, or median and range).<br>Data presented as mean ± SD; ≥3 independent experiments.<br>b. If applicable, the effect size with a confidence interval. No reported	Results 3
