# Supplementary material for: ASGR1 Deficiency Promotes Liver Fibrosis by Enhancing Pro‐Inflammatory Arachidonic Acid Metabolism and ALP Secretion
Source: Immun Inflamm Dis. 2026 Aug 20;14(8):e70496. doi: 10.1002/iid3.70496 (PMC13491154; doi:10.1002/iid3.70496)
Supplement: Supplementary file 2 [file IID3-14-e70496-s001.docx]

**ASGR1 Deficiency Promotes Liver Fibrosis by Enhancing Pro-inflammatory Arachidonic Acid Metabolism and ALP Secretion**

**Supplementary Information**

**Table S1. List of specific primers sequences used for qPCR analysis**

| Primer name | Primer Sequence (5’—3’) |
| --- | --- |
| Mice-F | TTTGAGAAGCCCTTGTCTTCCAGAG |
| Mice-R | ATGTGTCTGTGATCCTGAGCTGGGTA |
| Mice-WT/He-F | GGAACAGACTACGAGACAGGCTTCCA |
| Mice-GAPDH-F | GGAGAGTGTTTCCTCGTCCC |
| Mice-GAPDH-R | ATGAAGGGGTCGTTGATGGC |
| Mice-ATAC2-F | CCTGACTGAGCGTGGCTATT |
| Mice-ATAC2-R | AAGCGTTCGTTTCCAATGGTG |
| Mice-Col1a1-F | GCTTCACCTACAGCACCCTT |
| Mice-Col1a1-R | GGGTGGAGGGAGTTTACACG |
| Mice-Timp1-F | TTCTTGGTTCCCTGGCGTA |
| Mice-Timp1-R | CAAAGTGACGGCTCTGGTAG |
| Mice-Fn1-F | CGACTCTGACTGGCCTTACC |
| Mice-Fn1-R | ATGGCGTAATGGGAAACCGT |
| Mice-Tgfb1-F | AGATCGCCCTTTCATTTCAGAG |
| Mice-Tgfb1-R | GCAGTGGTAAACCTGATCCAGA |
| Mice-ALP-F | GGATATCGACGTGATCATGGGTG |
| Mice-ALP-R | GGAATGCTTGTGTCTGGTTT |
| Mice-ALP-R | GGAATGCTTGTGTCTGGTTT |
| Mice-18sRNA-F | CTCAACACGGGAAACCTCAC |
| Mice-18sRNA-R | CGCTCCACCAACTAAGAACG |
| Human-GAPDH-F | GGAGCGAGATCCCTCCAAAAT |
| Human-GAPDH-R | GGCTGTTGTCATACTTCTCATGG |
| Human-ATAC2-F | GTGTTGCCCCTGAAGAGCAT |
| Human-ATAC2-R | GCTGGGACATTGAAAGTCTCA |
| Hunan-ALP-F | AGATGACTACAGCCAAGGT |
| Human-ALP-R | CTCCACGAAGAGGAAGAAG |
| Rat-col1a1-F | GACTGTCCCAACCCCCAAAA |
| Rat-col1a1-R | TGGGTCCCTCGACTCCTATG |
| Rat-GAPDH-F | TTGTGCAGTGCCAGCCTC |
| Rat-GAPDH-R | ACCAGCTTCCCATTCTCAGC |
| Rat-ATAC2-F | GCTGCTCCAGCTATGTGTGA |
| Rat-ATAC2-R | AGTTGGTGATGATGCCGTGT |
| Rat-Fn1-F | CGGTGGCTGTCAGTCAAAG |
| Rat-Fn1-R | AAACCTCGGCTTCCTCCATAA |

**Abbreviations:** F, Forward sequence; R, Reverse sequence.

**Table S2. Antibodies used for Western blot and Immunohistochemistry**

| **Antibody name** | **Brand name** | **Lot number** |
| --- | --- | --- |
| Anti-α-SMA | Abcam | Ab124964 |
| Anti-COL1A1 | Bioss | PA2140-2 |
| HPR-conjugated GAPDH Monoclonal antibody | Proteintech | 60004-1-lg |
| HPR-Conjugated Alpha TUBULIN | Proteintech | 66031 |
| Anti-BAX | Proteintech | 2772 |
| Anti-ALP | Proteintech | 11187-1-AP |
| Anti-Alkaline Phosphatase, Tissue Non-Specific antibody | Abcam | Ab203106 |
| Alkaline Phosphatase (ALPL) Rabbit mAb | ABClonal | A0514 |
| Anti-ASGR1 | Proteintech | 11739-1-AP |
| HRP Horse Anti-Mouse IgG Polymer Detection Kit | Vector | MP-7402-15 |
| HRP Goat Anti-Rabbit lgG (H+L) | Life technologies | A0208 |
| Anti-GFAP | CST | 3670 |
| Donkey anti-Rabbit 488 | Life technologies | A21042 |
| Donkey anti-mouse 568 | Life technologies | A21134 |

**Figure S1.** A-C shows the results of total bilirubin amount (TBA), total bilirubin (T-Bil), direct bilirubin (D-Bil), and indirect bilirubin (I-Bil) in serum from WT, *Asgr1^+/-^* and *Asgr1^-/-^* t in the BDL-21d(A), CCl_4_-3w(B) and CCl_4_-6w.(C) experimental groups, respectively.

**Figure S2. Gene Expression of Pro-inflammatory and Pro-fibrotic Factors in ASGR1 knockout mice in CCI4-induced liver injury (3 weeks) and bile duct ligation (BDL-21day) models.** The mRNA expression of TGF, ALP, α-SMA, Fn1 in the three liver injury models. * P <0.05, ** P <0.01, **** P <0.0001.
